# Assessing the Peripheral Levels of the Neurotransmitters Noradrenaline, Dopamine and Serotonin and the Oxidant/Antioxidant Equilibrium in Circus Horses

**DOI:** 10.3390/ani14162354

**Published:** 2024-08-14

**Authors:** Raffaella Cocco, Sara Sechi, Maria Rizzo, Federica Arrigo, Claudia Giannetto, Giuseppe Piccione, Francesca Arfuso

**Affiliations:** 1 Department of Veterinary Medicine, University of Sassari, 07100 Sassari, Italy; rafco@uniss.it (R.C.); sarasechilavoro@tiscali.it (S.S.); 2 Department of Veterinary Sciences, University of Messina, 98168 Messina, Italy; federica.arrigo@studenti.unime.it (F.A.); claudia.giannetto1@unime.it (C.G.); gpiccione@unime.it (G.P.); farfuso@unime.it (F.A.)

**Keywords:** welfare, BAP, adrenergic system, circus, d-Roms, neurotransmitters, horse

## Abstract

**Simple Summary:**

It has been suggested that a circus has a limited ability to make improvements in animal welfare. Though the horse is a domesticated species, the activities as well as the management that horses are subjected to could perturb homeostasis, alerting the organism and resulting in stress conditions. In the current study, the plasma concentrations of noradrenaline, dopamine and serotonin, as well as the reactive oxygen metabolites (d-Roms) and the biological antioxidant potential (BAP), were assessed in horses managed in different circuses. According to the results herein found, it appears that the welfare of horses under circus management was not compromised; nevertheless, better attention to the management of old horses is advocated, as they could be more susceptible to oxidative stress.

**Abstract:**

Due to the paucity of information on circus management effects on the welfare of horses, this study investigated the plasma concentrations of noradrenaline, dopamine and serotonin, known to be indices of mental status, as well as the reactive oxygen metabolites (d-Roms) and the biological antioxidant potential (BAP), likely to denote the oxidant/antioxidant equilibrium of organisms, in horses managed in different Italian circuses. For the study, 56 circus horses of different breeds and ages were enrolled and divided into six groups according to the horses’ management (circus management, groups G1–G5; classic riding management representing the control group, CG). From each horse, blood samples were collected in order to assess the concentration of selected parameters. One-way ANOVA showed no differences (*p* > 0.05) in serotonin, dopamine, noradrenaline, d-Roms and BAP values between circus and control horses. No differences related to the breed of the horses enrolled in the study were found in the values of all investigated parameters (*p* > 0.05). Furthermore, neurotransmitters showed overlapping levels between the different age classes of investigated horses (*p* > 0.05); contrariwise, the age of the horse displayed a significant effect on BAP values, with the oldest horses (16–21 age class) exhibiting lower BAP values compared to 4–5, 6–10 and 11–15 age classes (*p* < 0.05), whereas the d-Roms showed similar values in horses of different age classes (*p* > 0.05). The results gathered in the present study suggest that the mental status of horses under circus management was not compromised; however, better attention and care in the management of older horses is advocated, as they showed a lower biological antioxidant potential than younger horses; thus, they could be more susceptible to oxidative stress.

## 1. Introduction

Since its domestication over 4000 years ago, the horse has been recruited for a variety of physical activities requiring extensive training to allow control by the rider, driver or handler. The resulting behavioral responses are supported by complex neural processes that facilitate learning. Horses are commonly enrolled in circus shows, and as in other work activities, they share with humans their work on a daily basis and have then “interpersonal” interactions not only with other working horses but also and mostly with a conductor who is the human who manages or rides them. Generally, a horse arrives at the circus around the age of three and become habituated to being around other horses and then to trusting his trainer. The daily physical and psychological gymnastics required to learn his trade will forge an athlete’s body, which will be able to respond to disturbances in homeostasis due to physical exercise with appropriate physiological responses related to various body systems such as the respiratory, cardiac, energetic and mental ones, to regain a state of balance. This skillful and patient training, always based on mutual respect between the trainer and the horse, enhances the latter’s natural attitudes and behaviors to make him an athlete circus horse [1]. Studies carried out on wild animals managed in circuses suggested that a circus has a limited ability to make improvements for animal welfare, due to restrictions in space and environmental enrichment, and their travelling nature, which necessitates frequent transportation [2,3,4]. To the best of the authors’ knowledge, no studies have focused on species-specific welfare implications for horses in a circus. Though the horse is a domesticated species, the activities as well as the management that horses are subjected to could perturb homeostasis, alerting the organism and resulting in a stress condition [5,6]. The capability of an animal to produce a suitable response to a stimulus that elicits a threat to homeostasis is crucial to survival. Therefore, a deep knowledge of functional adaptation of mammals throughout a stress condition is crucial for the monitoring of their health status and welfare. On the one hand, the response activated following a stressful event is positive for the organism, as it can be adaptive, leading to beneficial modification to brain regions supporting learning [6]; on the other hand, it could be maladaptive, causing welfare impairments and psychopathologies [6,7]. Moreover, the constant release of stress hormones by repeated stress is a characteristic sign of adverse health outcomes [8,9]. Indeed, it is widely acknowledged that a stress state leads to increase in reactive oxygen metabolites, resulting in damage at the cellular and tissue level, and it suppresses the immune competence of the animal, increasing the animal’s susceptibility to illnesses with negative consequences on performance and the animal’s welfare [8,9,10,11,12].

Oxidative stress must be considered especially in relation to the age of the animal, as it is known that older animals are more susceptible to oxidative stress, and whether it carries out physical activity. A study investigating the physiological stress response and health status of horses participating in the Sartiglia, a historical horse tournament held in Sardinia, Italy, based on the attempts of masked horsemen at a gallop to run a sword through a hole in a suspended silver star, showed that the tournament played an important role in causing high levels of oxidant markers not only because of the physical exercise but also because of the emotional stressors [13]. Endocrine variables may be useful as markers of well-being and fitness in the horse [13]. Welfare includes not only physical and behavioral components but also a psychological one [14,15,16]. The physiological stress response includes two interconnected branches of the autonomic nervous system: the rapidly responding sympathetic adrenal medullary (SAM) network, which leads to the adrenergic release of neurotransmitters into the blood and, ultimately, into the brain regions, and the slower hypothalamic pituitary adrenal (HPA) network, which leads to the release of glucocorticoid into the blood [17]. The activation of the adrenergic system leads to the “fight-or-flight” reaction by the release of neurotransmitters including noradrenaline, dopamine and serotonin. The activation of serotonin pathway enhances docility and friendliness, and, conversely, reduces aggression of the animal, whereas the noradrenergic system facilitates a number of anxiety-like behavioral responses [18]. This reaction is a natural mechanism to cope with daily novel stimuli which help animals, including horses, to adapt to their environments [18]. Furthermore, it is widely accepted that the noradrenergic system facilitates several anxiety-like behavioral responses including those induced by stress [19,20]. Nowadays, knowledge on the sympatho-adrenal responses and plasma neurotransmitters levels in horses managed in circus are missing in the scientific literature. Although no studies to date have been conducted on horses in circuses, the restrictions of space and environmental enrichment, as well as the frequent transport that horses must inevitably undergo due to the itinerant nature of circuses, can be stressful factors for the animals and therefore can affect their welfare. In view of the above considerations, the purpose of the current study was to investigate the plasma basal levels of some substances considered biomarkers related to welfare in domestics animals, and specifically linked to the mental status of animal, like noradrenaline, dopamine and serotonin in horses managed in different Italian circuses. Moreover, the reactive oxygen metabolites (d-Roms) and the biological antioxidant potential (BAP), likely to denote the oxidant/antioxidant equilibrium of organisms, were assessed. The study compares those levels with a control group to validate the similarities between horses used in different activities.

## 2. Materials and Methods

### 2.1. Animals and Study Design

The protocol of animal husbandry and experimentation were reviewed and approved in accordance with the standards recommended by the Guide for the Care and Use of Laboratory Animals and Directive 2010/63/EU for animal experiments. For the study, 56 circus horses of different breeds (Andalusian, Arabian, Pony and Friesian) and age (ranging from 4 to 21 years), with an average body weight of 389 ± 240 kg, were enrolled with the prior permission of their owners. As shown in Table 1, the study population was divided into 6 groups according to the horses’ management (circus management–groups G1–G5; classic riding management representing the control group, CG). Before starting the study, all animals underwent clinical examinations to assess body condition score (BCS), rectal temperature, heart rate, respiratory rate, appetite, and fecal consistency. All horses were subjected to routine biochemistry analyses (Table 2), and all were clinically healthy and free from internal and external parasites. The BCS was assessed on a 1–9 scale according to a previous study [10], with four horses presenting a BCS of 4 and fifty-two horses a BCS of 5.

The study was conducted at rest during a normal working day for the circus horses in January. The horses belonging to circus groups (G1–G5) and to the control group (CG) were kept in individual boxes (3.50 × 3.50 m), under natural environmental conditions (mean temperature 10.4 ± 3.3 °C; mean relative humidity 79%). The horses in groups G1–G5 were transported by road on average once a month along established routes. The horses belonging to the CG permanently lived in a riding stable, and they were not subjected to transportation. Moreover, the horses of CG were allowed to go to pasture during the day (from 10:00 AM to 4:00 PM). All animals were fed twice daily (6:00 AM and 6:00 PM). All horses received a total food amount of about 2.5% of their body weight in dry matter (forage:concentrate ratio 70:30), with water available ad libitum. All horses were taken out of their boxes daily in groups for exercise, training, and performances. The G1–G5 horses, in the morning, were exercised in a paddock and then trained inside the circus with the routines they would perform during shows (dressage). The training session consisted of three phases. A warm-up phase of 30 min of walk, trot and canter (~6 km/h); subsequently, the horses performed 11 min of specific exercises at a trot and at canter (halt, passage, piaffe, flying change, lateral movements, half pass, leg yield, shoulder-in, haunches-in, pirouette). All horses concluded with a 10 min recovery phase at a walk. All the horses participated in these sessions, including those that did not perform in the afternoon shows due to their age. The older horses were not required to perform any exercises but were kept in the group to maintain the position they had held throughout their lives. All horses in the control group were trained six days a week using the same exercises as the circus groups, by the same trainer and rider, respectively. During the clinical examination and blood sampling procedure, a veterinarian specialized in animal behavior observed all the horses enrolled in the study and did not find any behavioral problems.

### 2.2. Blood Sampling and Analysis

From each horse, blood samples were collected by jugular venipuncture into 8-mL vacutainer tubes with clot activator and 8-mL lithium heparin tubes (Terumo Co., Tokyo, Japan). All samples were taken in the boxes at rest (7:00 AM). Immediately after collection, blood samples were placed in refrigerated bags and transported to the laboratory for the analysis within three hours following sampling. Serum samples were obtained by centrifugation of blood collected into vacutainer tubes with clot activator; specifically, following standing at room temperature for 20 min, the tubes were centrifuged at 1600× *g* for 7 min at 16 °C and the serum concentration of serotonin, dopamine and noradrenaline was assessed by commercially available kits (Serotonin Research EIA Demeditec Diagnostics GmbH (Kiel, Germany), sensitivity of 0.005 ng/mL; Dopamine Plasma ELISA Demeditec Diagnostics GmbH (Kiel, Germany), sensitivity of 0.4 pg/mL; Noradrenaline EIA Demeditec Diagnostics GmbH (Kiel, Germany), sensitivity of 0.4 pg/mL). In plasma obtained by centrifugation of blood collected into lithium heparin tubes (1500× *g* for 90 s at 37 °C), the oxidant/antioxidant equilibrium was assessed by means of reactive oxygen metabolites (d-Roms; measured in Carratelli units [U Carr]) and biological antioxidant potential (BAP) tests (μmol/L) using the analytical system FRAS 4 Evolvo (H&D, Parma, Italy) according to a procedure previously used in horses [13].

### 2.3. Statistical Analysis

Data, expressed as mean values ± standard deviation, were tested for normality using the Shapiro–Wilk test. All data were normally distributed (*p* > 0.05), allowing for statistical analysis. One-way analysis of variance (ANOVA) was conducted to study the influence of the equestrian circus management on the studied parameters. When significant differences were found (*p* < 0.05), Dunnett’s post-hoc comparison test was applied to determine whether there were significant differences between circus and control horses. Additionally, all data were combined and reorganized, and one-way ANOVA was conducted to evaluate whether breed and age influenced the studied parameters. When significant differences were found (*p* < 0.05), Tukey’s post-hoc comparison test was applied. The data were analyzed with the software Prism v. 9.00 (GraphPad Software Ltd., San Diego, CA, USA, 2020).

## 3. Results

**Figure 1:**
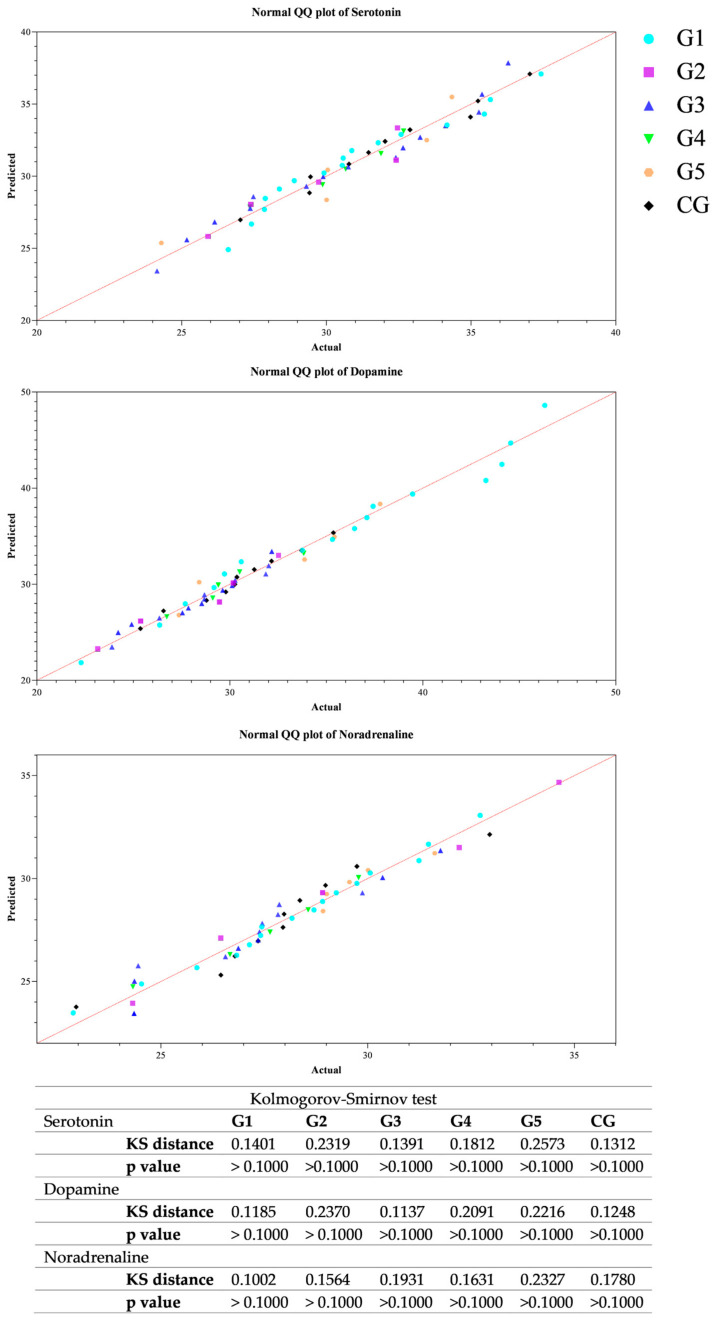
Normal quantile plot (Q-Q Plot) showing the normal distribution of serotonin, dopamine and noradrenaline values measured in investigated horses when analyzing the group’s effect (G1–G5 vs. CG).

**Figure 2:**
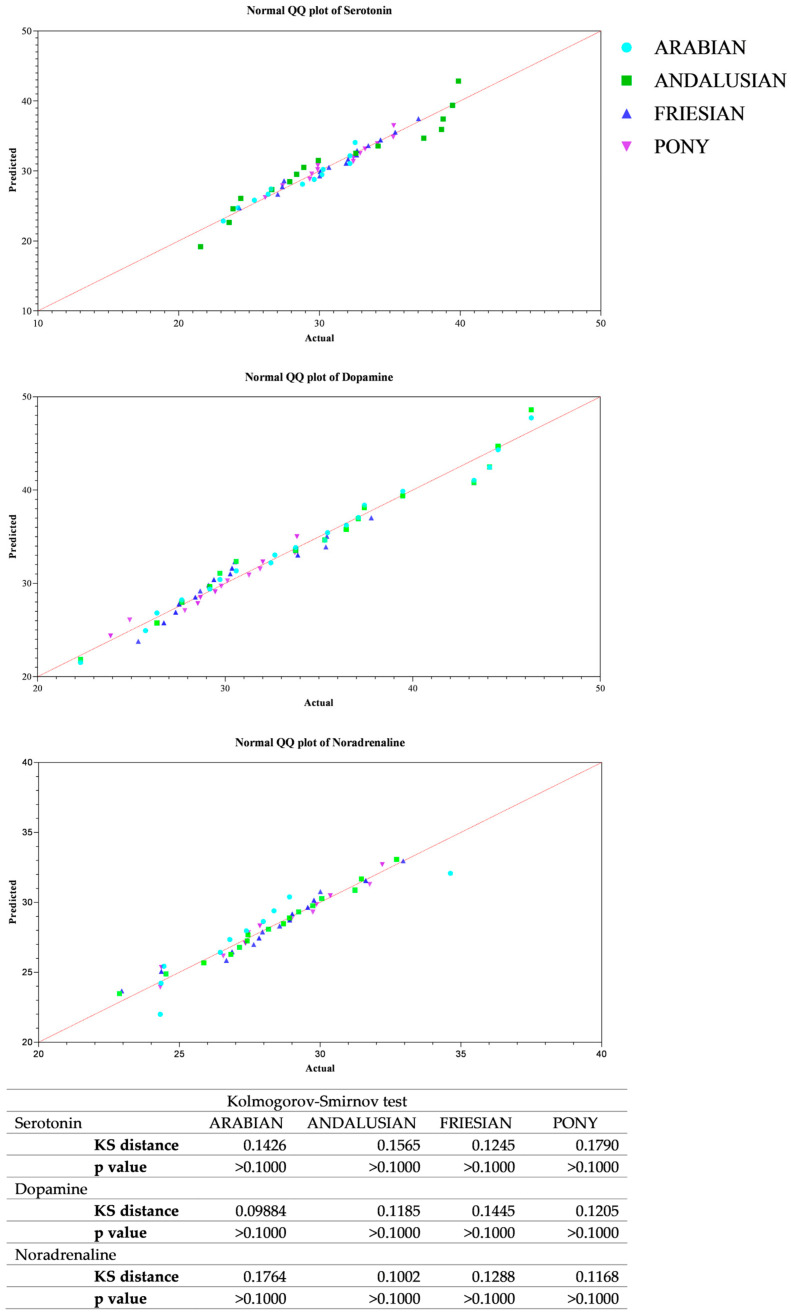
Normal quantile plot (Q-Q Plot) showing the normal distribution of serotonin, dopamine and noradrenaline values measured in investigated horses when analyzing the breed’s effect (Arabian, Andalusian, Friesian and Pony).

**Figure 3:**
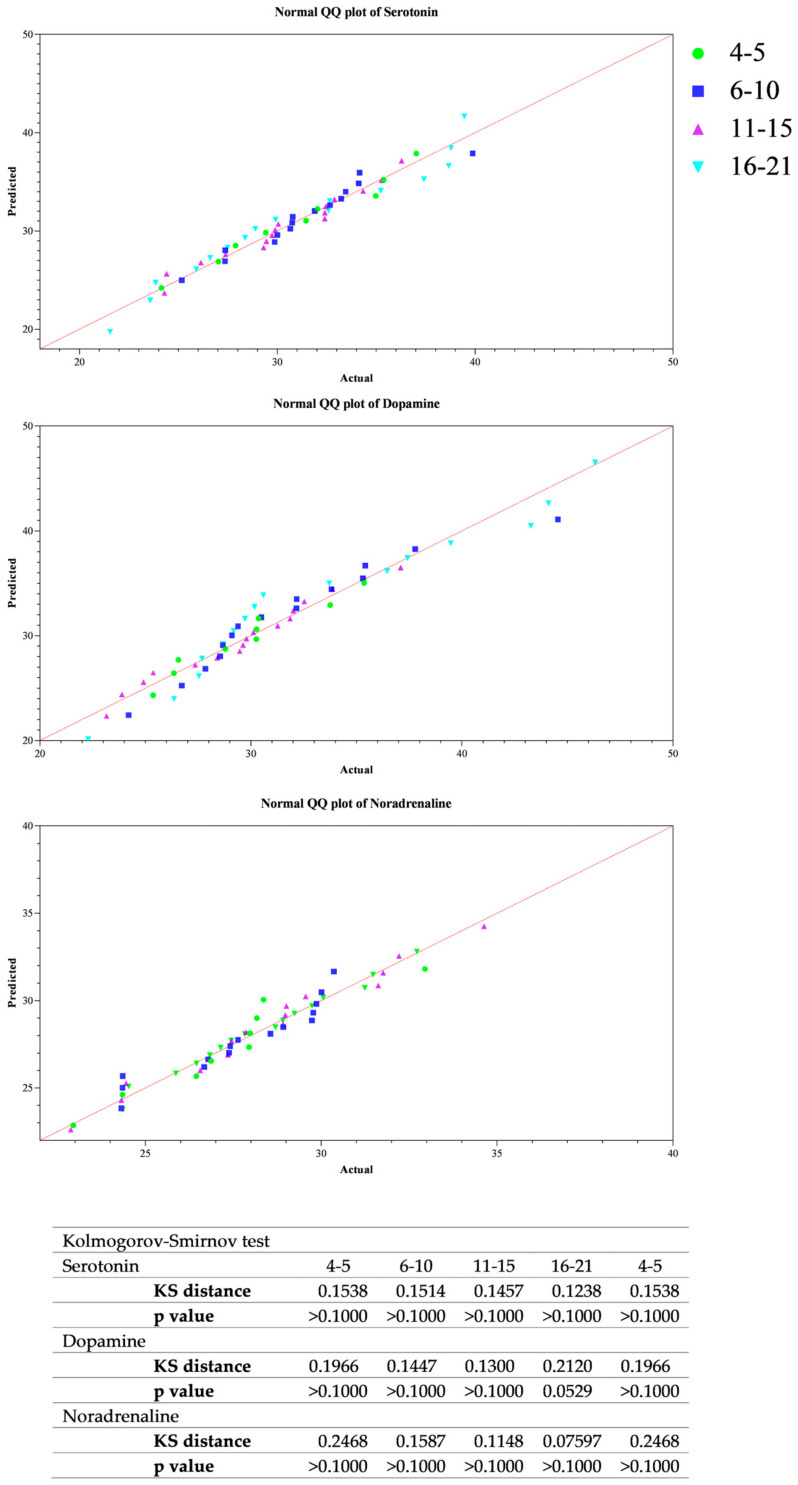
Normal quantile plot (Q-Q Plot) showing the normal distribution of serotonin, dopamine and noradrenaline values measured in investigated horses when analyzing the effect of age class (4–5, 6–10, 11–15 and 16–21 years).

**Figure 4:**
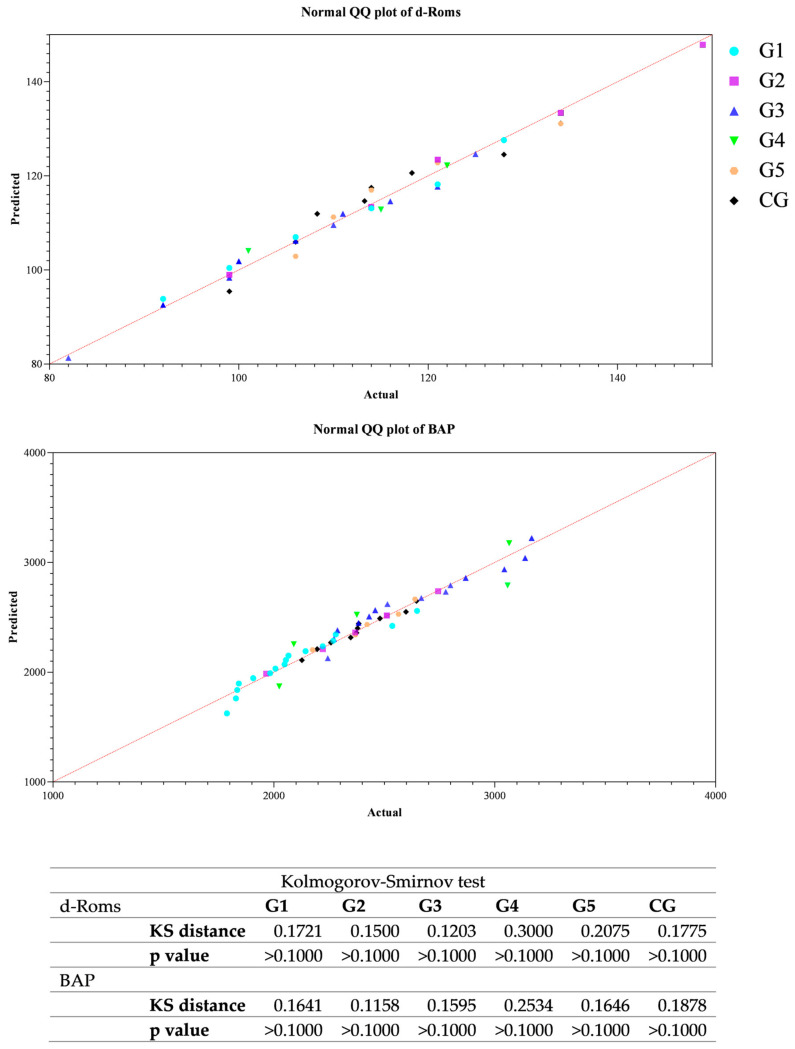
Normal quantile plot (Q-Q Plot) showing the normal distribution of the reactive oxygen metabolites (d-Roms) and the biological antioxidant potential (BAP) values measured in investigated horses when analyzing the group’s effect (G1–G5 vs. CG).

**Figure 5:**
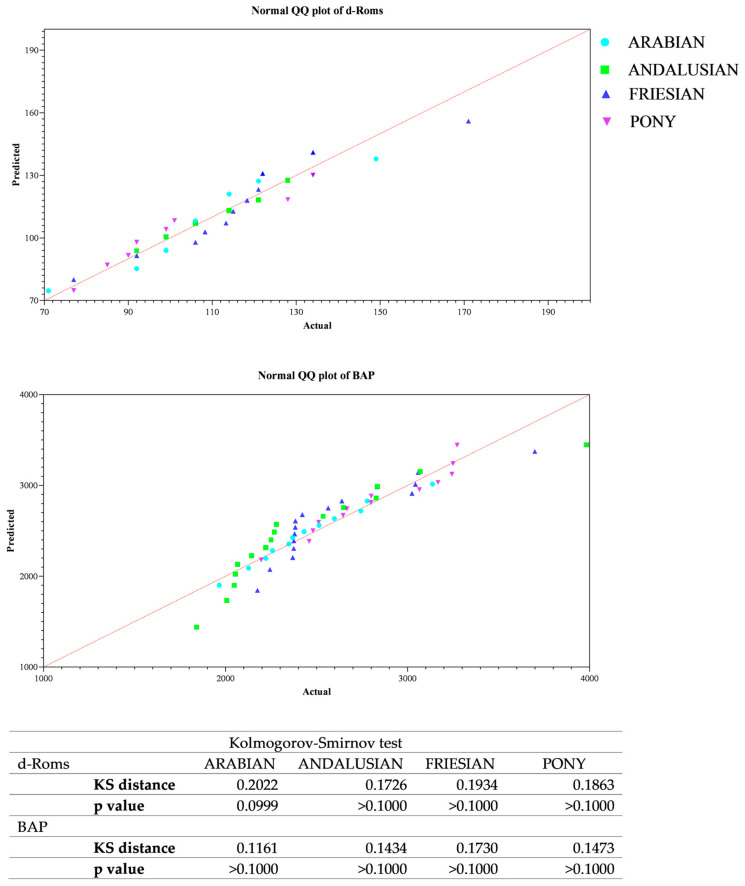
Normal quantile plot (Q-Q Plot) showing the normal distribution of the reactive oxygen metabolites (d-Roms) and the biological antioxidant potential (BAP) values measured in investigated horses when analyzing the breed’s effect (Arabian, Andalusian, Friesian and Pony).

**Figure 6:**
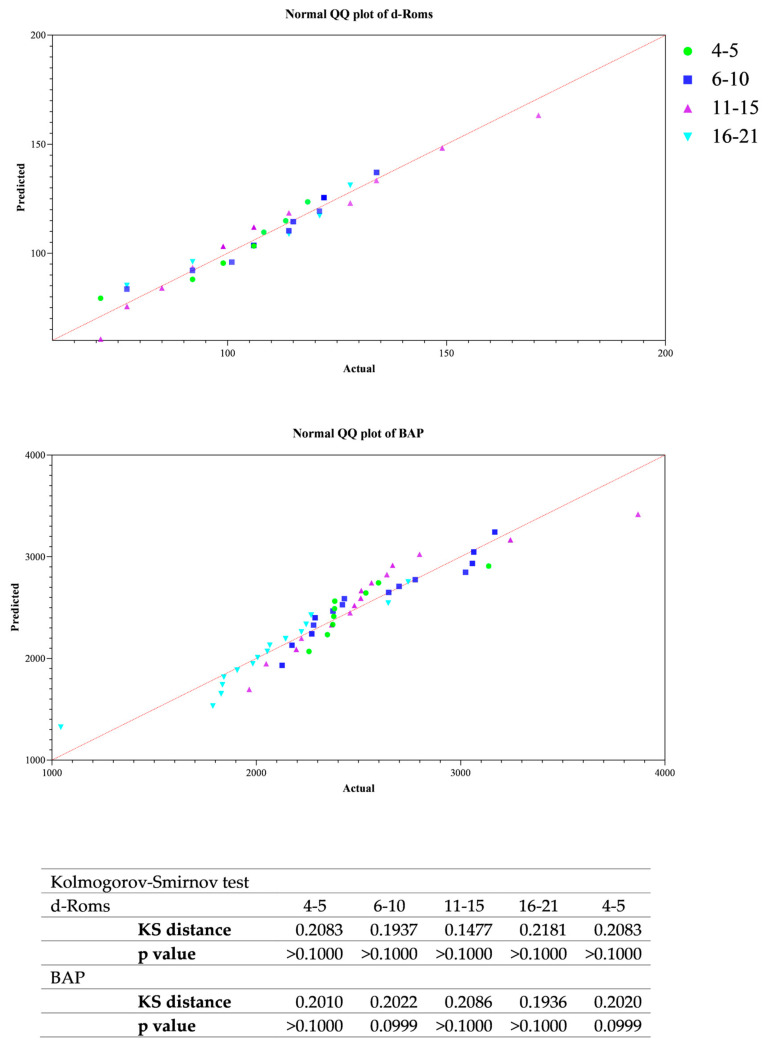
Normal quantile plot (Q-Q Plot) showing the normal distribution of the reactive oxygen metabolites (d-Roms) and the biological antioxidant potential (BAP) values measured in investigated horses when analyzing the effect of age class (4–5, 6–10, 11–15 and 16–21 years).

**Figure 7:**
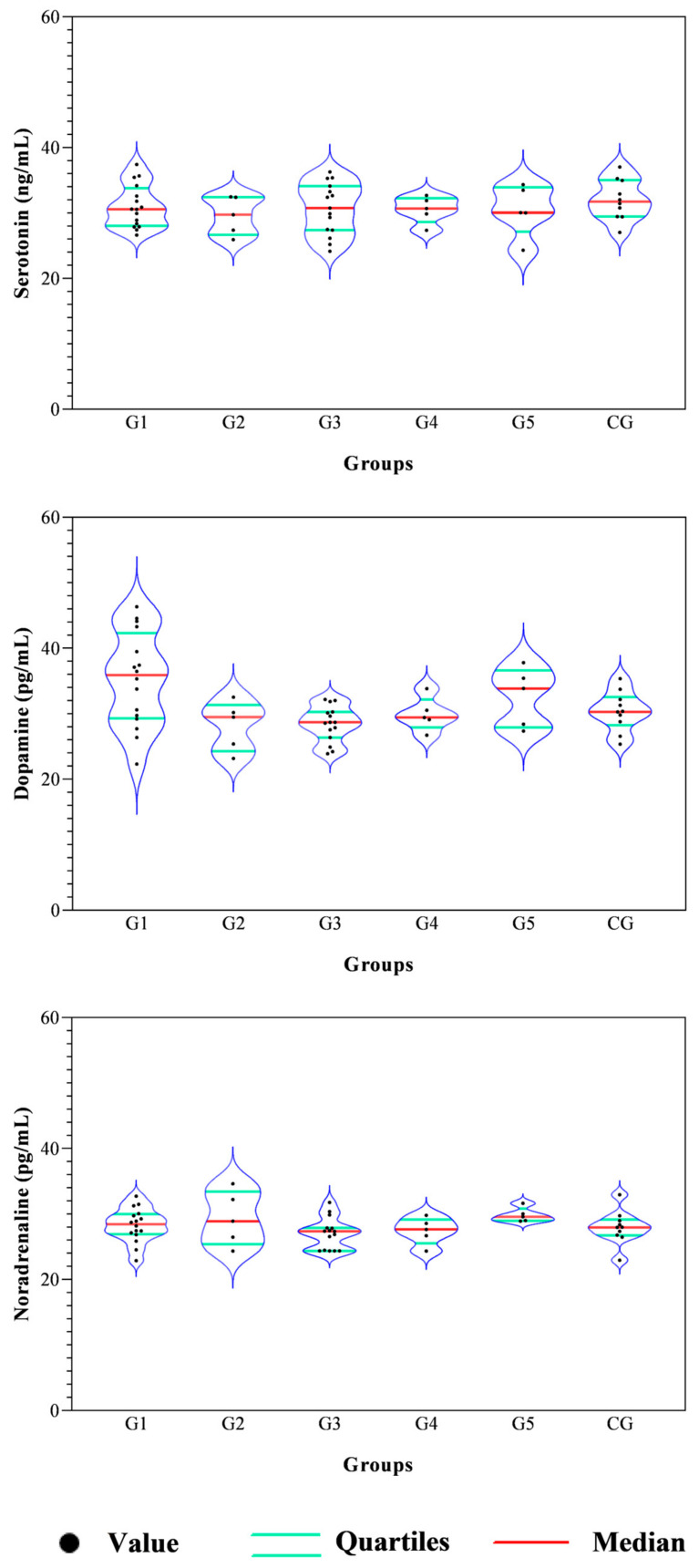
Violin plot showing distributions of serotonin, dopamine and noradrenaline values measured in investigated horses together with the relative statistical significances when analyzing the group’s effect (G1–G5 vs. CG).

**Figure 8:**
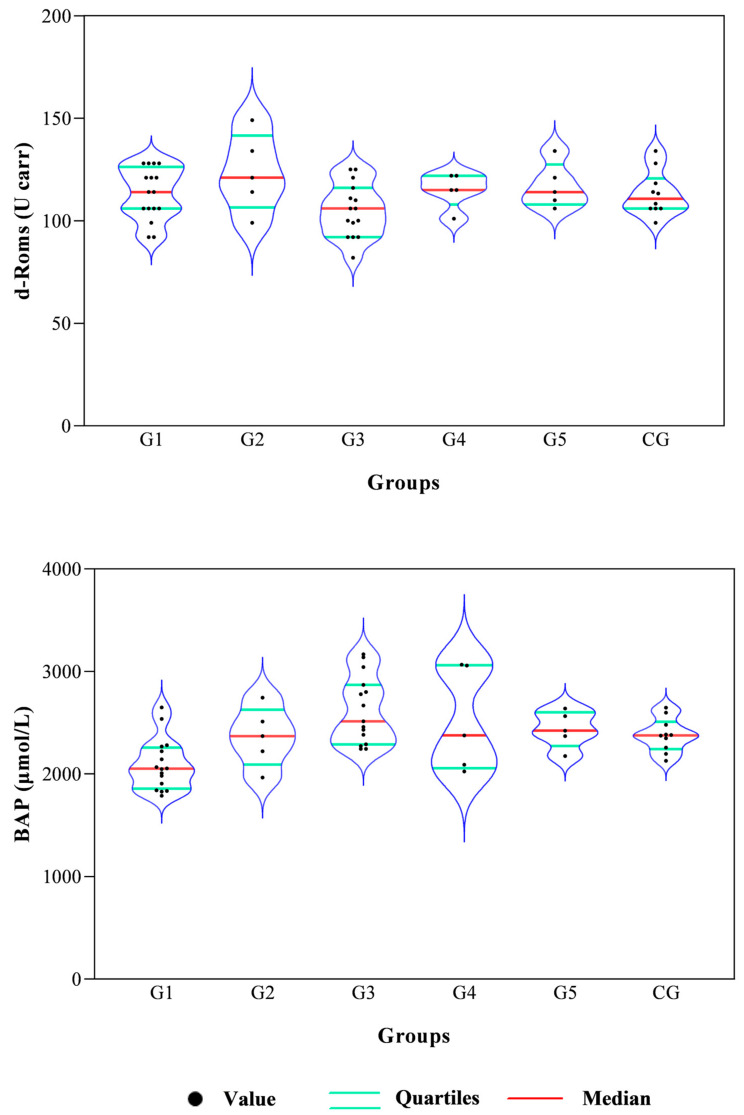
Violin plot showing distributions of the reactive oxygen metabolites (d-Roms) and the biological antioxidant potential (BAP) values measured in investigated horses together with the relative statistical significances when analyzing the group’s effect (G1–G5 vs. CG).

**Figure 9:**
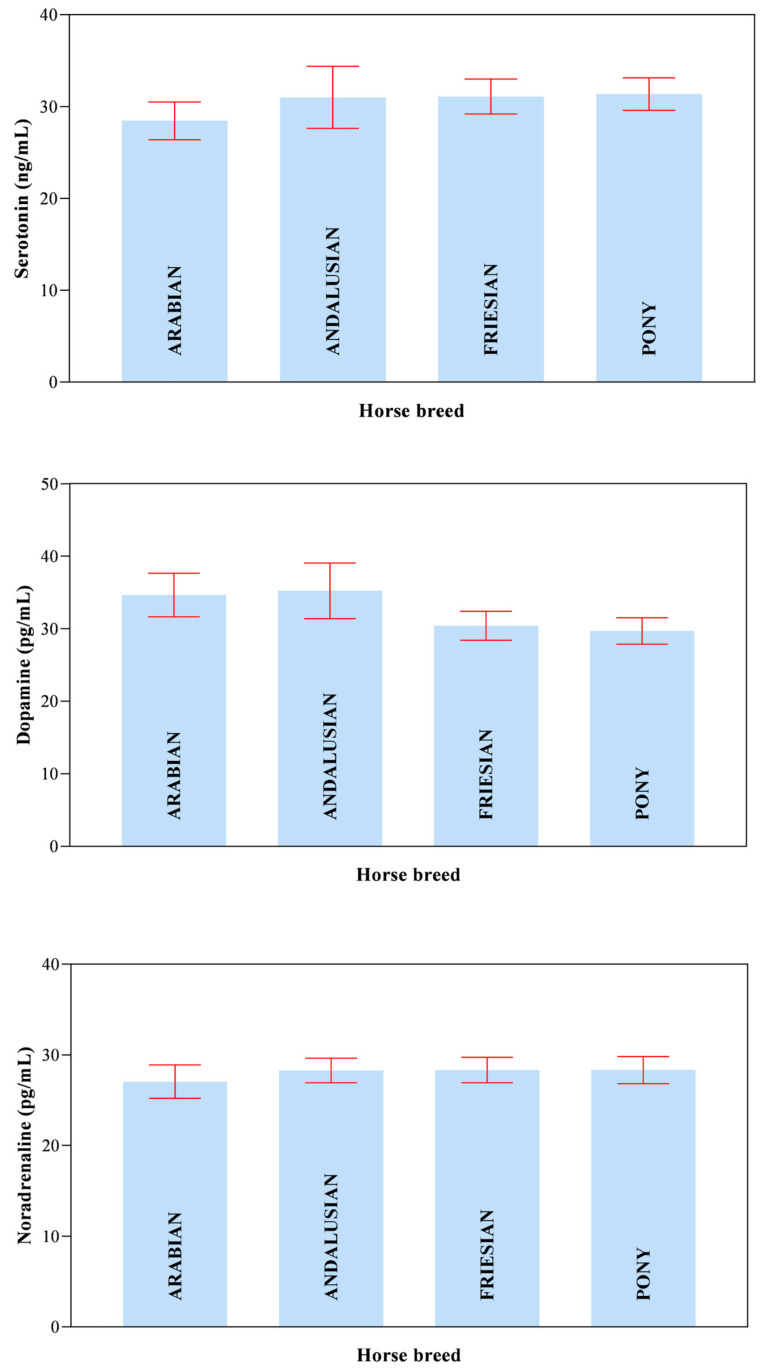
Mean values ± 95% confidence intervals of serotonin, dopamine and noradrenaline values measured in investigated horses together with the relative statistical significances when analyzing the breed’s effect (Arabian, Andalusian, Friesian and Pony).

**Figure 10:**
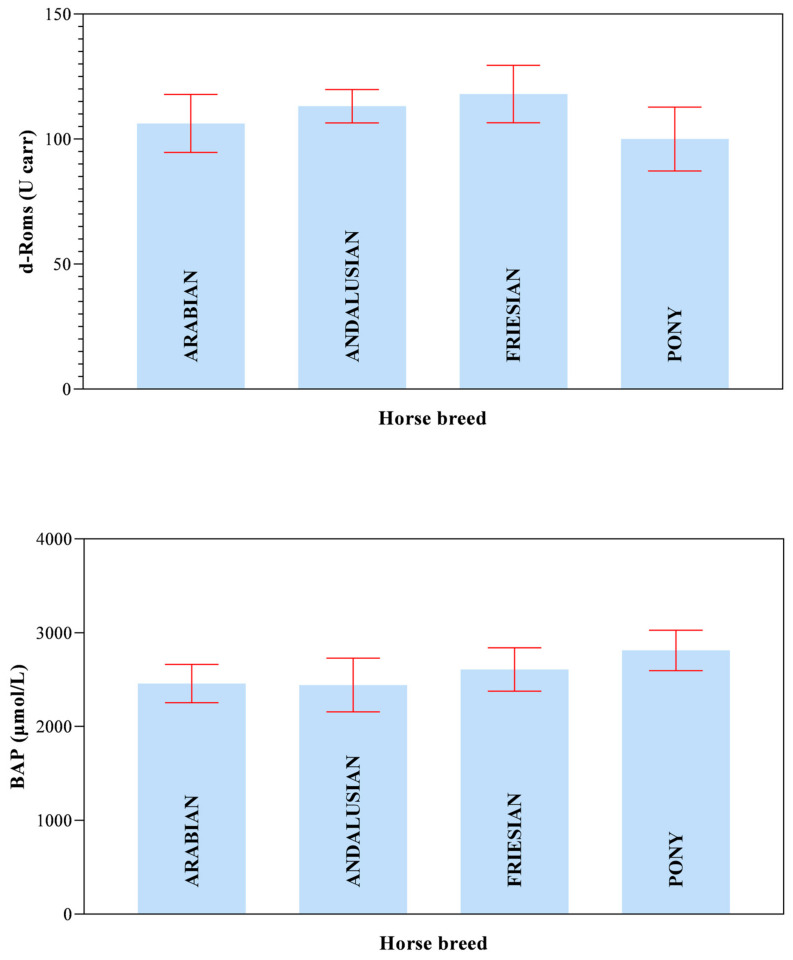
Mean values ± 95% confidence intervals of the reactive oxygen metabolites (d-Roms) and the biological antioxidant potential (BAP) values measured in investigated horses together with the relative statistical significances when analyzing the breed’s effect (Arabian, Andalusian, Friesian and Pony).

**Figure 11:**
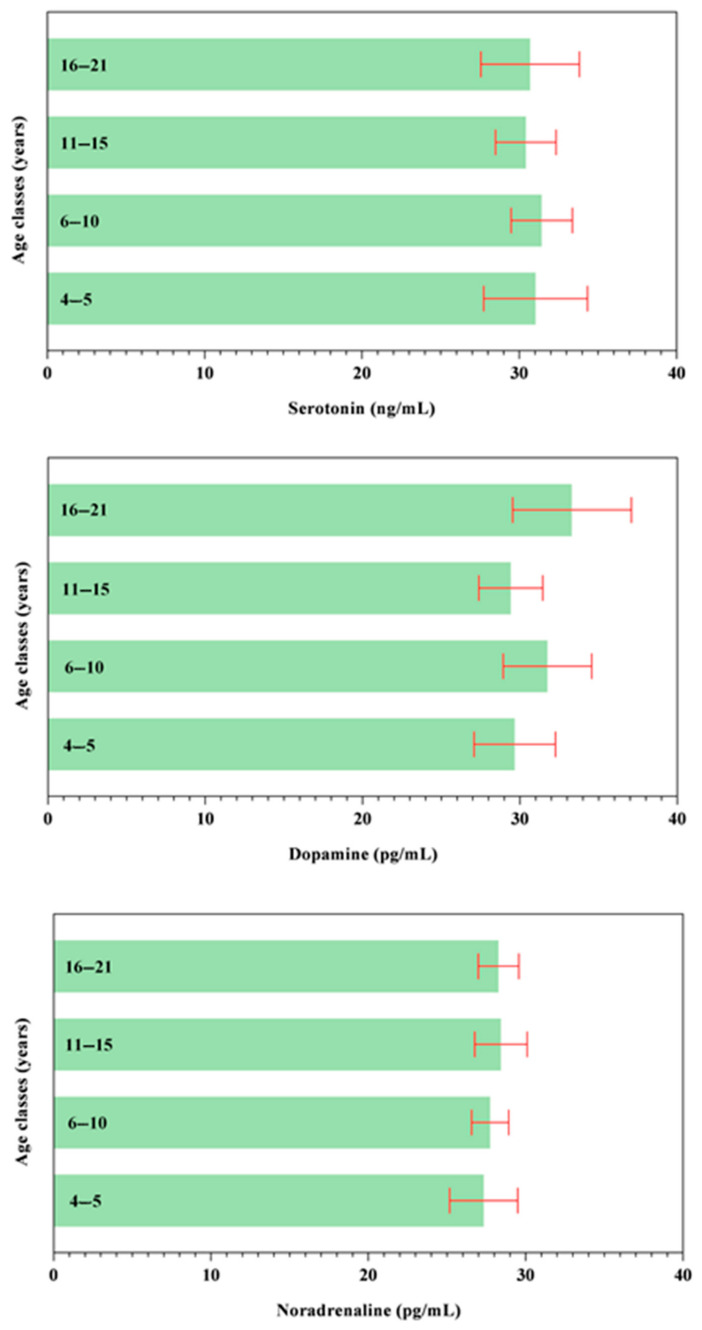
Mean values ± 95% confidence intervals of serotonin, dopamine and noradrenaline values measured in investigated horses together with the relative statistical significances when analyzing the effect of age class (4–5, 6–10, 11–15 and 16–21 years).

**Figure 12:**
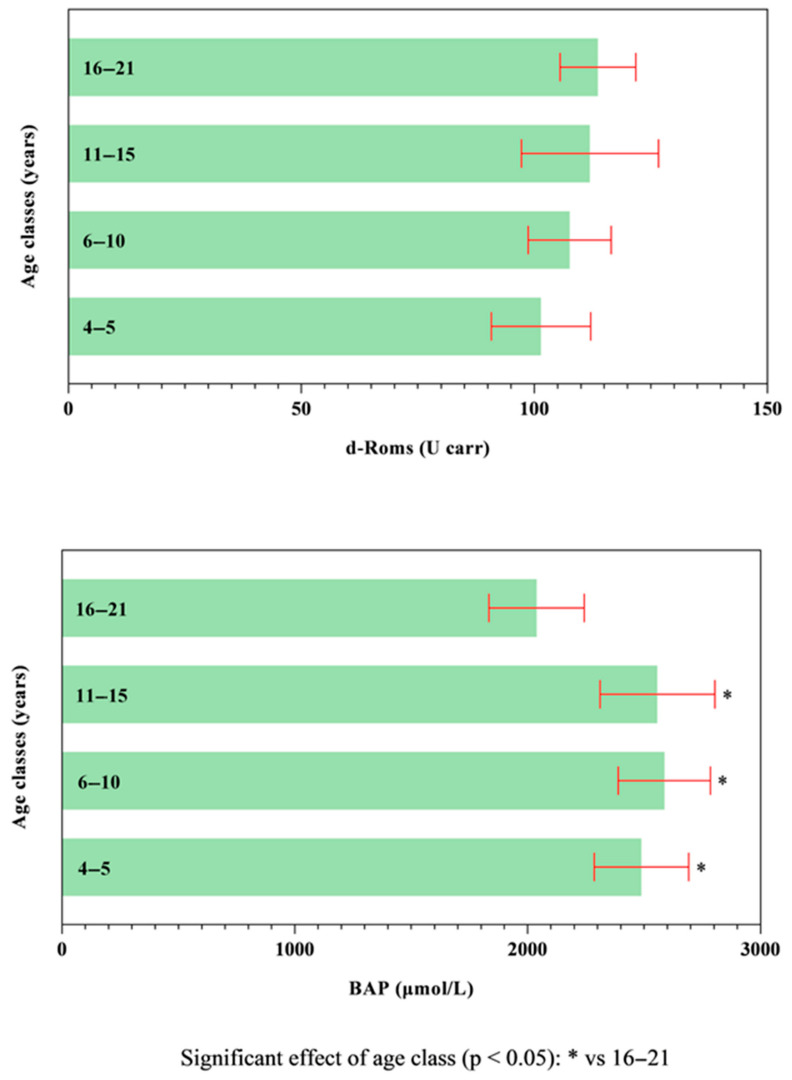
Mean values ± 95% confidence intervals of the reactive oxygen metabolites (d-Roms) and the biological antioxidant potential (BAP) values together with the relative statistical significances when analyzing the effect of age class (4–5, 6–10, 11–15 and 16–21 years).

All data passed a normality test, showing a normal distribution. Specifically, in Figure 1, Figure 2 and Figure 3 the normal distribution of the concentration of investigated neurotransmitters (serotonin, dopamine and noradrenaline) when analyzed among groups (G1–G5 vs. CG), breed and age classes was reported. In Figure 4, Figure 5 and Figure 6, the normal distribution of the d-Roms and BAP values when analyzed among groups (G1–G5 vs. CG), breed and age classes was shown. One-way ANOVA showed no statistically significant differences in neurotransmitter concentration (*p* > 0.05, Figure 7) and in the oxidant/antioxidant equilibrium (*p* > 0.05, Figure 8) assessed in circus horses (G1–G5) compared to the control group (CG). Considering the lack of significant differences between the groups (circus vs. control), the data were reorganized according to the breed (Arabian, Andalusian, Friesian and Pony) and age class (4–5, 6–10, 11–15 and 16–21 years) of the investigated horses. One-way ANOVA showed no statistically significant effect of breed on the concentration of neurotransmitters (*p* > 0.05, Figure 9) and on the oxidant/antioxidant equilibrium (*p* > 0.05, Figure 10). No statistically significant effect of age class on neurotransmitter concentration (*p* > 0.05, Figure 11) and on d-Roms (*p* > 0.05, Figure 12) was found in horses, whereas BAP values were influenced by the horses’ age, and Tukey’s post-hoc comparison test showed lower BAP values in the 16–21 age class than in the 4–5, 6–10 and 11–15 age classes (*p* < 0.05, Figure 12).

## 4. Discussion

There is a widespread belief that circuses have a negative impact on animal welfare domains such as nutrition, physical environment and health, mainly due to difficulties in obtaining appropriate veterinary care or nutrition and limitations in terms of size and complexity of temporary enclosures [21]. The paucity of scientific investigations carried out on domesticated animal species living in circus environments is one of the various causes of the lack of specific legislation or bans on circuses. To the best of authors’ knowledge, the current study investigated for the first time some aspects of welfare status in horses managed in circuses by assessing their plasma concentration of some neurotransmitters associated with mental status (i.e., serotonin, dopamine and noradrenaline) and their oxidant/antioxidant equilibrium. Since concentrations of these biomarkers of mental status may be influenced by time of sampling and by environment [22], in the current study blood sampling was performed under the same conditions for both circus and control horses. The choice of a morning sampling for all animals helped mitigate any potential influence of circadian rhythm on the concentration of parameters herein investigated [23]. According to the results gathered in the current study, all enrolled horses were clinically healthy, as they did not show clinical signs of disease and the parameters of the biochemical profile fell within the physiological reference ranges proposed for the equine species [22]. Furthermore, during clinical examination, all the animals did not present behavioral atypia and they proved to be calm during the blood sampling procedure without requiring any containment practice. The plasma concentration of all neurotransmitters (i.e., serotonin, dopamine and norepinephrine) as well as antioxidant oxidant balance parameters (i.e., d-Roms and BAP) studied in horses enrolled in this study passed the normality test, showing a Gaussian or normal distribution of the data when the effect of group (circus and control groups), as well as the effect of breed and age were analyzed. Regarding the effect of circus management, no parameter herein investigated showed significant differences between the horses belonging to the circus groups (G1–G5) and those belonging to the control group (CG). Noteworthy, the plasma concentration of serotonin, dopamine and noradrenaline, known to be indices of mental status, and the values of d-Roms and BAP, likely to denote the oxidant/antioxidant equilibrium of organism, overlapped between circus and control horses. It can be stated that the horses belonging to the control group were in an optimal management condition, as they were located under a good environmental enrichment, they could go out to pasture every day for at least 6 h, and during the week they worked only for 5 days a week with 2 days of rest. Furthermore, the horses were not subjected to transport. Therefore, the findings herein gathered seem to suggest that the mental status of horses under circus management was not compromised. Evaluating the animal mental status by means of non-invasive measurements of hormones is of increasing interest because this can serve as an effective tool to facilitate the optimization of environmental and husbandry conditions. Animals developed a physiological system to buffer themselves from adverse environmental conditions and to re-establish equilibrium. If external or internal stimuli are too strong or persist for a long time, they can be considered stressors eliciting a stress response. Apart from the slower HPA system response, the rapid-reacting SAM network leads to the release of neurotransmitters, including noradrenaline, dopamine and serotonin. In farm animals, circulating levels of these neurotransmitters provided information on the effects of acute environmental and psychological challenges [24]. As a matter of fact, the activation of the serotonin pathway improves the sociality of animals, thus enhancing docility and friendliness, and, conversely, reduces aggression [25]. Dopamine and noradrenaline, together with adrenaline, activate the adrenergic system involving the “fight or flight” reaction [26]. The noradrenergic system facilitates a number of anxiety-like behavioral responses, including those induced by stress, whether physiological or psychological [20,27]. However, a limitation of the current study is represented by the single sampling event, which is not a reliable basis on which to draw conclusions about longer-term responses to stress or potential welfare compromise, particularly in the case of the biomarkers chosen here. As a matter of fact, it has not been established in horses whether peripheral concentrations of these indicators reflect the effects of acute or chronic responses to environmental conditions. However, it is known that the neurotransmitters herein investigated are related to mental status; thus, it could be possible to speculate that horses used in circuses herein investigated have a normal mental status.

The values of serotonin, dopamine, noradrenaline, d-Roms and BAP did not show significant differences related to the breed of the horses enrolled in the study. Furthermore, neurotransmitters showed overlapping levels between the different age classes of investigated horses; contrariwise, the age of the horse displayed a significant effect on BAP values, with the oldest horses (in the 16–21 age class) exhibiting lower BAP values compared to the 4–5, 6–10 and 11–15 age classes, whereas the d-Roms showed similar values in horses of different age classes. The d-Roms values, used to measure the pro-oxidant activity, should be taken into account because some authors have indicated that in horses, high levels of free radicals increase the risk of laminitis [28] and respiratory diseases [29,30,31]. Contrariwise, the BAP concentration provided a measure of the antioxidant capacity of the animal, as it counteracts the simultaneous increase in the oxidative metabolites, as pro-oxidative imbalance can influence the onset of many diseases and pathological conditions caused by an abnormal amount of free radicals in body cells [32]. The lower BAP found in oldest horses could be related to the ageing process. However, this hypothesis disagrees with previous findings [33] showing that age had no significant effect on antioxidant parameters in clinically normal horses. Though aging is not a disease by itself, it makes the organism more vulnerable to many disorders [34,35]. Most age-related diseases are associated with a low level of chronic inflammation [36,37], and the oxidative stress, due to up-regulation of free radical formation and the overall reduction in cellular antioxidant capacity, has been recognized to play a major role in determining and maintaining the low-grade inflammation observed in aging and age-associated diseases [36,37]. The results of the current study suggest that the investigated horses, even the older ones, were not in a state of oxidative stress at the enrolment, as the d-Roms values were similar to those of the younger horses; however, the lower BAP values measured in the older horses seem to suggest greater attention and care in the management of old horses, as they are more susceptible to oxidative stress.

## 5. Conclusions

A solid scientific framework on welfare condition of horses subjected to circus management is missing in literature so far. The current study provides a first scientific approach assessing the basal peripheral plasma concentrations of some neurotransmitters associated with mental states as well as the oxidant/antioxidant equilibrium in horses managed in different Italian circuses. Though a small population of horses managed in circuses was evaluated with a single blood sampling and results should be interpreted with caution, the results gathered in the present study seem to suggest that the horses involved in circus activities were in a normal mental status and they were adapted physiologically to their management condition. However, according to the findings herein obtained, better attention and care in the management of older horses is advocated, as they showed a lower biological antioxidant potential than younger horses; thus, they could be more susceptible to oxidative stress. Additional prospective studies should be performed to collect more objective, evidence-based data in a greater number of horses managed in circus by including also more blood sampling time points and by considering further indicators of good mental and behavioral states to provide a more holistic view of their welfare state.

## Figures and Tables

**Table 1:** Signalment data for enrolled horses divided into groups based on their respective circus (G1–G5) and control group (GC).

Groups	Breed	N	Age	Gender	N
**G1**		**16**			
	Andalusian	16	Median 16 years	Geldings	12
			Range 4–21	Females	4
**G2**		**5**			
	Arabian	4	Median 14 years	Geldings	4
	Pony	1	Range 12–18	Females	1
**G3**		**15**			
	Pony	8	Median 11 years	Geldings	10
	Arabian	4	Range 4–21	Females	5
	Friesian	3			
**G4**		**5**			
	Friesian	5	Median 8 years	Geldings	5
			Range 6–8	Females	0
**G5**		**5**			
	Friesian	4	Median 13 years	Geldings	5
	Pony	1	Range 8–14	Females	0
**GC**		**10**			
	Arabian	4	Median 5 years	Geldings	6
	Pony	3	Range 4–18	Females	4
	Friesian	3			

**Table 2:** Mean ± standard deviation of serum biochemical parameters (total proteins, TP; albumin; creatinine; urea; aspartate aminotransferase, AST; alanine aminotransferase, ALT; alkaline phosphatase, ALP; γ-glutamyltransferase, GGT; total bilirubin; cholesterol; triglyceride, calcium, Ca; phosphorus, P) measured in investigated horses belonging to circus groups (G1–G5) and control group (GC).

Serum Biochemical Parameters	G1 (n = 16)	G2 (n = 5)	G3 (n = 15)	G4 (n = 5)	G5 (n = 5)	GC (n = 10)
Total protein (g/dL)	6.39 ± 0.41	6.50 ± 0.32	6.33 ± 0.39	7.12 ± 0.40	6.32 ± 0.86	6.21 ± 0.61
Albumin (g/dL)	3.60 ± 0.21	3.18 ± 0.52	3.49 ± 0.23	3.51 ± 0.53	2.83 ± 0.42	3.07 ± 0.51
Creatinine (mg/dL)	1.26 ± 0.05	1.42 ± 0.16	1.45 ± 0.14	1.56 ± 0.45	1.24 ± 0.19	1.62 ± 0.71
Urea (mg/dL)	26.56 ± 3.69	26.40 ± 9.89	26.73 ± 2.63	32.20 ± 2.49	23.00 ± 5.15	26.86 ± 3.28
AST (U/L)	252.80 ± 21.31	229.00 ± 74.24	280.80 ± 33.42	306.00 ± 61.76	302.80 ± 77.41	282.70 ± 49.26
ALT (U/L)	14.25 ± 1.48	14.00 ± 1.23	12.47 ± 6.52	10.80 ± 4.60	11.40 ± 4.56	12.14 ± 5.78
ALP (U/L)	151.60 ± 9.44	153.00 ± 6.48	159.60 ± 4.11	217.60 ± 17.44	231.00 ± 10.00	167.40 ± 10.10
GGT (U/L)	11.19 ± 2.04	11.60 ± 2.30	10.47 ± 1.17	8.00 ± 1.45	10.40 ± 1.10	8.56 ± 1.81
Total bilirubin (mg/dL)	1.32 ± 0.20	1.26 ± 0.38	1.23 ± 0.15	1.50 ± 0.43	1.38 ± 0.31	1.38 ± 0.36
Cholesterol (mg/dL)	74.88 ± 7.97	75.00 ± 6.06	77.60 ± 6.12	79.00 ± 7.88	79.00 ± 7.18	79.43 ± 4.47
Triglycerides (mg/dL)	19.75 ± 6.09	19.80 ± 4.21	23.20 ± 3.08	21.00 ± 4.18	22.60 ± 3.78	23.14 ± 3.73
Ca (mg/dL)	12.75 ± 0.38	13.54 ± 0.95	12.22 ± 0.55	12.64 ± 0.99	12.56 ± 0.50	12.06 ± 0.41
P (mg/dL)	2.71 ± 0.47	3.18 ± 0.48	3.22 ± 0.51	12.72 ± 2.83	3.10 ± 0.33	3.06 ± 0.59

## Data Availability

Data supporting reported results can be found in this manuscript.
